# Association of genetic polymorphisms with laryngeal carcinoma prognosis in a Chinese population

**DOI:** 10.18632/oncotarget.14381

**Published:** 2016-12-30

**Authors:** Fang Quan, Feipeng Zhang, Yanxia Bai, Long Zhou, Hua Yang, Bin Li, Tianbo Jin, Huajing Li, Yuan Shao

**Affiliations:** ^1^ Department of Otolaryngology & Head Neck, The First Affiliated Hospital of Xi’an Jiaotong University, Xi’an, Shaanxi, 710061, China; ^2^ Sichuan Yanting Middle School, Mianyang, Sichuan, 621600, China; ^3^ School of Life Sciences, Northwest University, Xi’an, Shaanxi, 710069, China; ^*^ These authors have contributed equally to this work

**Keywords:** laryngeal carcinoma, single-nucleotide polymorphism, overall survival, hazard ratio

## Abstract

We analyzed the effects of single-nucleotide polymorphisms (SNPs) on laryngeal carcinoma (LC) risk and overall survival (OS) in 170 Chinese male LC patients followed for 10 years. After assessment of clinical characteristics (age, laryngectomy, neck dissection, tumor differentiation, TNM status), the patients were genotyped for 24 SNPs associated with risk in multiple cancers. LC risk was assessed using log-rank test and Cox proportional hazard models. The median OS time was 48 months. By the follow-up deadline, OS was 41.2%. Kaplan-Meier analysis indicated 1-, 3-, and 5-year survival rates to be 84.7%, 57.2%, and 47.1%, respectively. Five LC clinicopathological characteristics, namely total laryngectomy (TL), low differentiation (LD), T3-T4, N1-N2, and clinical stage III-IV were associated with worse OS (HR: 2.35, *p* < 0.001; HR: 2.39, *p* = 0.02; HR: 2.17, *p* < 0.001; HR: 2.39, *p* < 0.001; and HR: 3.29, *p* < 0.001, respectively). Univariate cox regression analysis indicated that four SNPs were associated (*p* < 0.05) with LC OS in the codominant genetic model compared to patients with the homozygous wild-type genotype: rs10088262 G/A (HR = 1.57), rs1665650 A/G (HR = 0.65); rs3802842 C/C (HR = 2.18), and rs59336 T/A and T/T (HR = 0.61 and 2.61, respectively).

## INTRODUCTION

Laryngeal carcinoma (LC) is a common tumor of the head and neck. It accounts for 1% - 5% of all malignant tumors, comprises 3.3% - 8.1% of all the head and neck malignant tumors, and among these its incidence is only lower than that of nasopharyngeal carcinoma [1]. According to GLOBOCAN 2012 [2], 156,877 individuals were diagnosed with LC worldwide, while 20,014 cases were diagnosed in China. In this country, the age-adjusted incidence was 1.1 per 100,000, and the age-adjusted mortality rate was 0.7 per 100,000 for both sexes. LC generally affects people aged 50-70 years, especially males. In China, the ratio of men to women diagnosed with LC was 10.5:1 [2, 3]. In recent years, the incidence of LC has increased steadily because of multiple carcinogenic factors [4, 5], and in China is predicted to reach 55,900 new cases/year in the next 5 years. As with other cancers, the pathogenesis of laryngeal carcinoma involves the combined effects of environmental and genetic factors. To date, several genes have been implicated in the occurrence of LC and have been shown to affect its prognosis [6–8]. Recent studies have shown that single genes may be associated with many related cancers [9]. For example, the murine double minute 2 (*MDM2*) gene has been proposed to contribute to the emergence and development of many tumors, especially digestive carcinomas [10] and LC [1].

The association of single nucleotide polymorphisms (SNP), the most common form of genetic variation, with multiple cancers, has also been highlighted [11, 12]. While extensive studies have evaluated the relevance of clinicopathological parameters such as surgical treatment modalities, age, tumor stage, differentiation, and lymph node metastasis, as prognostic factors in head and neck cancers [13], only a few basic studies have revealed an essential role of specific genes in digestive tract cancers. Moreover, the association between SNPs within or near these genes and LC prognosis has not been fully investigated. In the present study, we analyzed the association of 24 SNPs related to digestive tract cancers with LC prognosis in 170 Han Chinese male patients, and identified four SNPs significantly associated with OS. These results shed light on the genetic component of LC and may prove useful to guide further studies addressing its pathogenesis.

## RESULTS

### Patient, tumor, and treatment characteristics

170 LC male patients with a mean age of 60.7 years (range, 32 to 82 years) were enrolled in this study; none of them presented distant metastases (i.e they were M0). According to primary tumor and lymph node stage, 40 cases (23.5%) were T1, 62 cases (36.5%) were T2, 50 cases (29.4%) were T3, and 18 cases (10.6%) were T4, while 116 cases (68.2%) were N0, 30 cases (17.6%) were N1, and 24 cases (16.1%) were N2. Follow-up time, survival status, tumor stage and age distribution are shown in Table 1. 37 patients underwent neck dissection and 133 patients had non-neck dissection. Surgical procedures are listed in Table 2.

**Table 1:** Clinicopathological characteristics of the patients included in this study

Patient Characteristics	No.	%
Total	170(male, M0 ^c^)	100
Min follow-up time (month)	3	-
Max follow-up time (month)	122	-
Median follow-up time (month)	38	-
Survival status	70 survivors	41.2
100 dead	58.8	
Mean Age	60.75	
Range	32-82	
<60	80	47.1
≥60	90	52.9
Tumor Stage ^a^		
T1	40	23.5
T2	62	36.5
T3	50	29.4
T4	18	10.6
N0	116	68.2
N1	30	17.6
N2	24	14.1
Clinical Stage ^b^		
I	37	21.8
II	36	21.2
III	61	35.9
IV	36	21.2
Differentiation degree		
Low	14	8.2
Moderate	125	73.5
High	31	18.2
Merged Surgical Procedures		
Neck Dissection	37	21.8
Non-Neck Dissection	133	78.2

a: T-stage: tumor stage; N-stage: lymph node stage

b: Clinical stage reference: international unifying new TNM classification from Union for International Cancer Control

c: M0: No distant metastasis;

**Table 2:** Univariate Cox proportional hazards analysis of potential factors affecting survival

Variable	Wald	HR (95% CI)	p ^a^	Details	Median		1-, 3-, 5-year survival rate	Overall comparison		
					Estimate	95%CI		Chi-Square	df	*p* ^b^ for log-rank
Total					48.00	29.26-66.74	0.929, 0.788, 0.671			
Age	0.55	1.16 (0.78-1.72)	0.460	<60	59.00	30.05-87.95	0.85, 0.584, 0.414			
				≥60	48.00	29.86-66.14	0.867, 0.633, 0.507	0.556	1	0.456
Laryngectomy	17.64	2.35 (1.58-3.49)	<0.001*	PL	73.00	50.84-95.16	0.885, 0.73, 0.632			
				TL	30.00	21.32-38.68	0.818, 0.468, 0.27	18.96	1	<0.001*
Neck dissection	1.69	0.71 (0.43-1.19)	0.194	ND	36.00	-	0.676, -, -			
				NND	56.00	36.32-75.69	0.91, 0.752, 0.583	1.73	1	0.188
Differentiation	8.99		0.011*	HD	71.00	15.55-126.45	0.676, -, -			
	0.07	0.93 (0.57-1.57)	0.794	MD	59.00	39.14-78.86	0.912, 0.712, 0.602			
	5.44	2.39 (1.15-4.99)	0.02*	LD	15.00	0.00-33.33	0.214, -, -	9.78	2	0.008*
T	14.07	2.17 (1.45-3.25)	<0.001*	T1-T2	77.00	56.18-97.82	0.882, 0.695, 0.616			
				T3-T4	32.00	22.25-41.75	0.824, 0.483, 0.243	14.96	1	<0.001*
N	17.04	2.39 (1.58-3.62)	<0.001*	N0	71.00	48.28-93.73	0.897, 0.706, 0.6			
				N1-N2	26.00	14.08-37.92	0.796, 0.403, -	18.33	1	<0.001*
Clinical stage	26.86	3.29 (2.10-5.18)	<0.001*	I-II	98.00	67.49-128.51	0.849, 0.732, 0.527			
				III, IV	32.00	24.2-39.8	0.897, 0.649, 0.418	30.15	1	<0.001*

a. *p* for Wald test < 0.05 indicates statistical significance for individual coefficients

b. *p* for Log-rank Test < 0.05 indicates statistical significance for grouping variables

*Abbreviations:* PL: Partial laryngectomy; TL: Total laryngectomy; ND: Neck dissection; NND: Non-neck dissection; HD: High differentiation; MD: Moderate differentiation; LD: Low differentiation; df: degrees of freedom. *: indicates statistical significance

### Overall survival analysis

At the median follow-up period of 38 months (range, 3 to 122 months), the mean and median survival times were 62.13 and 48 months, respectively. By the follow-up time deadline, OS was 41.2% (100 dead and 70 survivors). Kaplan-Meier statistical analysis indicated that 1-, 3-, and 5-year survival rates were 84.7%, 57.2% and 47.1%, respectively [14].

### Analysis of clinical characteristics

We performed univariate Cox proportional hazards analysis to evaluate the association of age, laryngectomy, neck dissection, differentiation, T stage, N stage, and clinical stage with LC survival rates. Significant correlations were found for laryngectomy (HR: 2.35, 95% CI: 1.58-3.49, Wald-*p* < 0.001); tumor differentiation (HR: 2.39, 95%CI: 1.15-4.99, Wald-*p* = 0.02); T stage (HR: 2.17, 95% CI: 1.45-3.25, Wald-*p* < 0.001); N stage (HR: 2.39, 95% CI: 1.58-3.62, Wald *p* < 0.001); and clinical stage (HR: 3.29, 95% CI: 2.10-5.18, Wald*-p* < 0.001). The log-rank test further validated the significance of these five variables (Table 2). Total laryngectomy (TL) median survival time (30 months; 95% CI: 21.32-38.68) was significantly shorter than that of partial laryngectomy (PL; 73 months; 95% CI: 50.84-95.16; log-rank *p* <0.001) [14]. With respect to tumor differentiation status, a significant difference was detected between low differentiation (LD) and high differentiation (HD) groups (log-rank *p* = 0.008): median survival time was 71 months (95% CI: 15.55-126.45) for HD, 59 months (95% CI: 39.14-78.86) for moderate differentiation (MD), and 15 months (95% CI: 0.00-33.33) for LD [14]. Stratification based on primary tumor staging showed a significantly longer median survival time of 77 months (95% CI: 56.18-97.82) for T1-T2, compared to 32 months (95% CI: 22.25-41.75) for T3-T4 (log-rank *p* < 0.001) [14]. Lymph node staging analyses also showed a significant difference in the median survival time of N0 (71 months; 95% CI: 48.28-93.73) versus N1-N2 (26 months; 95% CI: 14.08-37.92; log-rank *p* < 0.001) [14]. Additionally, clinical stage (TNM status) subgroup analysis revealed a significant longer median survival time for stage I-II (98 months; 95% CI: 67.49-128.51) compared to III-IV (32 months; 95% CI: 24.2-39.8; log-rank *p* < 0.001) [14].

### SNP analysis

**Figure 1:**
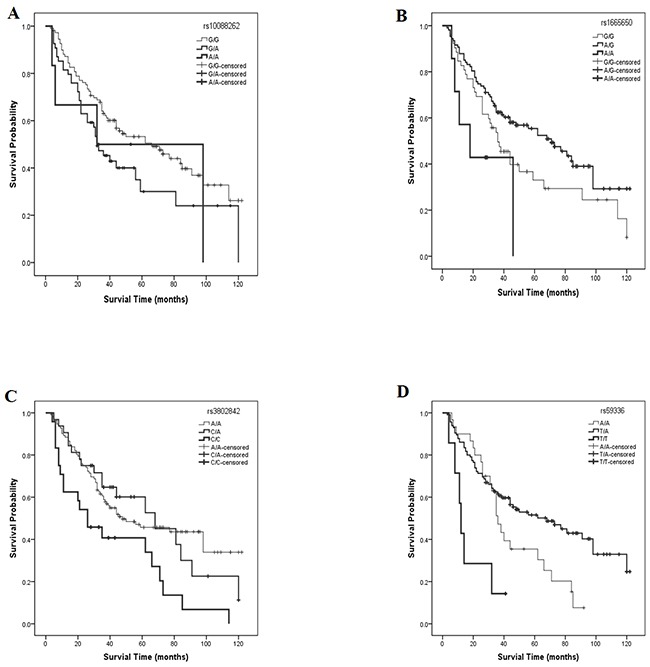
Survival rate curves for different SNP polymorphisms

In SNP univariate analyses, the lower frequency allele was coded as the ‘risk’ allele. All SNP genotypes were coded as 0, 1, or 2, to represent the number of risk alleles they possessed for that SNP. The HR and 95% CI of levels 1 and 2 were compared with those for level 0 (reference genotype). Preliminary results showed significant differences for four SNPs, namely rs10088262, rs1665650, rs3802842 and rs59336 (Table 3 and Figure 1A). Among these, a significant overall effect on survival was detected for three SNPs. The median survival times for patients with rs1665650 genotypes 0, 1 or 2 were 36, 71, and 18 months (χ^2^ = 18.96, log-rank *p* < 0.001) respectively (Figure 1B); for rs3802842 genotypes 0, 1 or 2, median survival times were 48, 68, and 26 months (χ^2^ =10.06, log-rank *p* = 0.007) respectively (Figure 1C); for rs59336 genotypes 0, 1 or 2, median survival times were 36, 68, 12 months (χ^2^ = 15.21, log-rank *p* < 0.001) respectively (Figure 1D).

**Table 3:** Analysis of SNPs associated with OS in LC patients

SNP	Genotype	Total N	Variables in the Equation				Median	95% CI	Chi-Square	*p* ^b^ for log-rank
			Wald	HR	95% CI	*p* ^a^				
rs10088262	G/G (0)	55	4.69			0.096	66.00	39.47-92.53		
	G/A (1)	71	4.38	1.57	1.03-2.39	0.036*	32.00	22.64-41.36		
	A/A (2)	34	0.76	1.57	0.57-4.34	0.382	32.00	0-87.21		
	Overall	160					48.00	30.56-65.44	4.84	0.809
rs1665650	G/G (0)	52	7.99			0.018*	36.00	25.73-46.27		
	A/G (1)	107	3.95	0.65	0.43-0.99	0.047*	71.00	49.22-92.78		
	A/A (2)	7	2.00	1.98	0.77-5.09	0.157	18.00	0.04-35.94		
	Overall	166					48.00	30.54-65.46	8.56	0.014*
rs3802842	A/A (0)	112	9.44			0.009*	48.00	18.72-77.29		
	C/A (1)	32	0.01	1.02	0.60-1.73	0.939	68.00	31.09-104.91		
	C/C (2)	24	9.03	2.18	1.31-3.61	0.003*	26.00	9.00 -43.00		
	Overall	168					50.00	31.06-68.94	10.06	0.007*
rs59336	A/A (0)	30	13.3			0.001*	36.00	30.31-41.69		
	T/A (1)	115	4.09	0.61	0.37-.985	0.043*	68.00	38.56-97.44		
	T/T (2)	7	4.28	2.61	1.05-6.49	0.039*	12.00	9.43-14.57		
	Overall	152					46.00	27.65-64.35	15.21	<0.001*

*p* ^a^ for Wald test < 0.05 indicates statistical significance for individual coefficients

*p* ^b^ for Log-rank Test < 0.05 indicates statistical significance for grouping variables

*: indicates statistical significance

### Multivariate Cox proportional hazards analysis

We performed multivariate Cox regression analysis by including the five clinical predictors that showed statistical significance in univariate analyses. Results indicated that two SNPs (rs10088262 and rs3802842) correlated significantly with OS in LC patients: compared with “GG” in rs10088262, the risk rate of genotype “G/A” was 1.969 (95% CI: 1.26-3.09, *p* = 0.003); on the other hand, the risk rate of rs3802842 (C/C vs. A/A) was 1.839 (95% CI: 1.10-3.08, *p* = 0.021) (Table 4).

**Table 4:** Multivariate Cox proportional hazards analysis of rs10088262 and rs3802842 adjusted for HD, MD, LD, T stage and clinical stage

SNP	Variable	Wald	df	HR	95% CI	*p* ^a^
rs10088262	rs10088262(G/G)	8.853	2			0.012*
	rs10088262(G/A)	8.719	1	1.969	1.26-3.09	0.003*
	rs10088262(A/A)	0.661	1	1.534	0.55-4.29	0.416
rs3802842	rs3802842(A/A)	7.014	2			0.030*
	rs3802842(C/A)	0.510	1	0.820	0.48-1.41	0.475
	rs3802842(C/C)	5.353	1	1.839	1.10-3.08	0.021*

*Abbreviations:* HD: High differentiation; MD: Moderate differentiation; LD: Low differentiation; df: degrees of freedom

*: indicates statistical significance.

## DISCUSSION

Our study analyzed, during a ten-year follow-up, the prognostic association of clinical parameters and multiple cancer-related SNPs in 170 male LC patients from northwest China. We found that five clinical characteristics [laryngectomy, tumor differentiation, tumor status (T), regional lymph node status (N), and clinical (TNM) stage] were correlated with survival. Specifically, clinical outcome was affected by laryngectomy variants, as TL had poorer prognosis than PL, with a median survival of 30 months, compared with 73 months for PL. This conclusion, however, may be confounded by the fact that TL is more often performed to remove larger malignant tumors, which carry higher risk. The associations found for other clinical features were consistent with the findings of previous studies, as low tumor differentiation, and higher T, N, and clinical stages all correlated with worse prognoses [15, 16].

Of the 24 SNPs analyzed, four showed an association with LC prognosis in univariate analyses. After adjusting for significant clinical parameters, multivariate regression analysis revealed that two SNP genotypes, i.e. “G/A” of rs10088262 (HR: 1.969) and “C/C” of rs3802842 (HR: 1.839) were significantly associated with LC prognosis. The SNPs analyzed here have been shown to be associated with risk for diverse types of cancer. Rs10088262 is an intergenic variant located on chromosome 8q24.13; its minor allele “A” has been correlated with reduced risk of esophageal cancer [12]. However, in the present study the genotype “G/A” of this SNP indicated poor prognosis, with a median survival time of 32 months, compared with 66 months for the “G/G” genotype.

Carrying the “C” allele at rs3802842 has been associated with a lower risk for rectal tumors [17]. Another study, however, suggested that the “CC” genotype of rs3802842 may significantly increase colorectal cancer (CRC) risk in the recessive model [18]. Our analysis revealed that the median survival of genotype “C/C” of the rs3802842 polymorphism was only 26 months, significantly less than the homozygous wild genotype “A/A” (48 months). Rs3802842 maps to a region between “colorectal cancer associated 1” and “colorectal cancer associated 2” (*C11orf92/COLCA1*- *C11orf93/COLCA2*) genes, located on 11q23.1, which are arranged on opposite strands and share a regulatory region that contains genetic variants that are in high linkage disequilibrium with rs3802842. Expression levels of *COLCA1* and *COLCA2* transcripts correlate with rs3802842 genotypes. Genetic, expression and immunohistochemical data implicate *COLCA1* and *COLCA2* in the pathogenesis of colon cancer, whereas histologic analyses indicate the involvement of immune pathways [19].

## MATERIALS AND METHODS

### Patients and methods

### Study population

170 male patients, aged 32-82 years (average age, 60.7 years), were enrolled at the First Affiliated Hospital of Xi’an Jiao Tong University from January 30, 2002 to April 7, 2003; patient follow-up ended on April 7, 2013. Patients received neither radiotherapy nor chemotherapy before enrollment, and in all cases LC diagnosis was confirmed by two pathologists. All participants were unrelated Han Chinese and had no other malignancy histories.

### Patient demographics and blood collection

A standardized epidemiological questionnaire including residential region, age, smoking status, alcohol use, ethnicity, education status, and family history of cancer was used to collect personal information through in-person interviews. Related information was collected through a consultation with the treating physicians or from medical chart reviews. LC staging relies on the TNM system designed jointly by the Union International Cancer Control Version 7.0 (UICC 7.0). Venous blood samples (5 ml) and signed informed consent were obtained from each participant. All blood samples were quickly frozen in liquid nitrogen and stored at -80°C. This study was approved by the ethics committee of the First Affiliated Hospital of Xi’an Jiao Tong University.

### SNP selection and genotyping

Using the HapMap database, 24 candidate SNPs with minor allele frequencies > 5% in the Asian population and previously published associations with other cancers were selected from chromosomes 8, 9, 10, 11, and 12. Basic information about the 24 SNPs is listed in Table 5. Genomic DNA was extracted from peripheral blood using phenol–chloroform, and its concentration was measured using a DU530 UV/VIS spectrophotometer (Beckman Instruments, Fullerton, CA, USA) according to the manufacturer’s protocol. MassARRAY Assay Design 3.0 Software (Sequenom, San Diego, California, USA) was used to design Multiplex SNP MassEXTEND assays[20]. Genotyping was performed using the Sequenom MassARRAY RS1000 following a standard protocol recommended by the manufacturer [20], and data were analyzed using Sequenom Typer 4.0 Software (Sequenom, San Diego, CA, USA) [20, 21].

**Table 5:** SNPs analyzed

SNP	Band	A/B	Gene	Cancer type	Ref
rs2439302	8p12	C/G	NRG1	Thyroid	[22]
rs7832232	8p11.22	G/A	intergenic	Pancreatic	[23]
rs10088262	8q24.13	A/G	intergenic	Esophageal	[12]
rs10505477	8q24.21	T/C	intergenic	Gastric	[24]
rs6983267	8q24.21	G/T	intergenic	Prostate	[25]
rs7014346	8q24.21	A/G	POU5F1B	Colorectal	[26]
rs13294589	9p21.2	G/A	intergenic	Esophageal	[12]
rs10114408	9q22.32	T/A	intergenic	Colorectal	[27]
rs965513	9q22.33	A/G	intergenic	Thyroid	[22]
rs10795668	10p14	A/G	intergenic	Colorectal	[28]
rs2274223	10q23.33	G/A	PLCE1	Esophageal	[29]
rs1665650	10q25.3	A/G	HSPA12A	Colorectal	[30]
rs12413624	10q26.11	T/A	intergenic	Colorectal/Gastric	[31]
rs10500715	11p15.4	G/T	SBF2	Pancreatic	[32]
rs3824999	11q13.4	C/A	POLD3	Colorectal	[33]
rs3802842	11q23.1	A/C	C11orf92-C11orf93	Colorectal	[18]
rs10774214	12p13.32	T/C	intergenic	Colorectal	[34]
rs3217901	12p13.32	A/G	CCND2	Colorectal	[34]
rs10879357	12q21.1	G/A	TPH2	Colorectal	[34]
rs671	12q24.12	A/G	ALDH2	Esophageal	[35]
rs4767364	12q24.13	G/A	NAA25	Aero-digestive tract	[36]
rs11066280	12q24.13	A/T	C12orf51	Gastric	[37]
rs59336	12q24.21	T/A	TBX3	Colorectal	[34]
rs7315438	12q24.21	T/C	intergenic	Colorectal/Esophageal	[38]

A/B stands for minor/major alleles on the sample frequencies.

### Statistical analysis

Patients’ baseline characteristics, disease stage, and treatment modalities were summarized using descriptive statistics. The overall survival (OS) time was defined as the period from diagnosis until death of any cause or until the date of the last follow-up, at which data point was censored. All summary statistics on time-to-event variables were estimated according to the Kaplan-Meier method and compared using the log-rank test. Univariate and multivariate Cox proportional hazards regression models were used to calculate the hazard ratios (HR), and 95% confidence intervals (95% CI) of the effect of clinical variables and SNPs, respectively, on the overall survival (OS) of LC patients. SPSS software (version 21.0) was used for statistical analysis. A *p* value < 0.05 was considered significant.

## CONCLUSION

We found that five clinicopathological charac-teristics, namely total laryngectomy, low differentiation, T3-T4, N1-N2, and clinical stage III-IV, were associated with survival in LC patients. Although four SNP were found to be significantly associated with OS in univariate cox regression analysis, multivariate analysis showed that two SNPs (rs10088262 and rs3802842) were associated with LC prognosis after adjustment for clinical factors. Combined with previous research, our study suggests an association for these SNPs with multiple cancers. Further larger studies are required to validate our findings and to assess the molecular mechanisms underlying the observed associations.
